# Influence of Overheating Phenomenon on Bitumen and Asphalt Mixture Properties

**DOI:** 10.3390/ma12040610

**Published:** 2019-02-18

**Authors:** Michał Sarnowski, Karol J. Kowalski, Jan B. Król, Piotr Radziszewski

**Affiliations:** Faculty of Civil Engineering, Warsaw University of Technology, 00-637 Warsaw, Poland; k.kowalski@il.pw.edu.pl (K.J.K.); j.krol@il.pw.edu.pl (J.B.K.); p.radziszewski@il.pw.edu.pl (P.R.)

**Keywords:** bitumen, asphalt mixture, overheating, SBS

## Abstract

In the course of manufacturing, transport and installation, road bitumens and asphalt mixtures can be exposed to the impact of elevated process temperatures exceeding 240 °C. This mainly applies to the mixtures used for road pavements and bridge deck insulation during adverse weather conditions. The heating process should not change the basic and rheological properties of binders and the asphalt mixtures that to a degree cause the degradation of asphalt pavement durability. The work involved analyzing the properties of non-modified bitumens and SBS polymer modified bitumens, heated at temperatures of 200 °C, 250 °C and 300 °C for 1 h. Next, the asphalt mixtures were heated in the same temperatures. Based on the developed Overheating Degradation Index (ODI) it was demonstrated that polymer-modified bitumens were characterized by higher overheating sensitivity A_(ODI)_ than non-modified bitumens, which was confirmed by mixture test results. Overheating limit temperatures T_(ODI)_ were determined, which in the case of polymer-modified bitumens are up to 20 °C lower than for non-modified bitumens. When the temperature increases above T_(ODI)_, loss of viscoelastic properties occurs in the material which causes, among other effects, a loss of resistance to fatigue cracking.

## 1. Introduction

Bituminous binders and asphalt mixtures during technological processes (production, transport, application) can be exposed to high temperatures, greatly exceeding the permissible temperature determined by a binder manufacturer or in the domestic technical documentation. An example of a mixture which is particularly exposed to the impact of overheating is mastic asphalt (MA), which is commonly used in Europe for the construction of bridge pavements. For example, according to the Polish requirements of the General Directorate for National Roads and Motorways [1], the highest temperature of mastic asphalt cannot exceed 230 °C when it is produced with bitumen 35/50. The limit temperature for the MA mixture with other binders, like modified bitumens, was not determined. Decreased workability of mastic asphalt compared to other asphalt mixtures and the need to conduct the work in adverse weather and process conditions (wind, thin layer, bridge pavement layer cooling from the top and bottom), make it possible for the mastic asphalt during transport and maturation in boilers to maintain workability and to be heated for over 1 h, even at a temperature of 240 °C. The phenomenon of overheating occurs also in the case of other asphalt mixtures, such as asphalt concrete (AC) and stone mastic asphalt (SMA), the application of which in the case of bridges is common. Another reason for overheating of bitumen may be an emergency situation, for example a bridge fire, fire in a tunnel or a car on fire standing on an asphalt pavement. The third possible reason for overheating may be excessive heating of the pavement during renovation works in hot technology (e.g., operation of radiators, heaters, etc.).

An efficient method to eliminate the phenomenon of overheating of asphalt materials during production and paving is the application of Fischer-Tropsch wax additives, which enables application mixtures in the “warm mix” technology (Warm-Mix Asphalt) [2,3]. However, the “hot mix” technology is applied in most cases. A too high temperature acting on an asphalt mixture is destructive, mainly to bituminous binders, which can impact pavement durability.

Research on the impact of the overheating phenomenon on the properties of binders and mixtures is conducted globally to a very limited extent. Numerous studies [4,5,6,7,8,9,10,11] concerning the evaluation of excessive temperature impact on bitumen composites are limited to analyzing ageing phenomena ongoing at process temperatures, which are below 190 °C. The impact of additional, high temperature on bitumen and an asphalt pavement can support the self-healing process [12,13]. However, this applies to pavement reconstruction technology, which is already used, and the applied temperature does not exceed values typical for the asphalt technology. The general knowledge in the field of the asphalt overheating phenomenon is often limited to a conclusion that this process may cause oxidation and stiffening of the binder [14]. The sensitivity to temperature and time- and temperature behavior of bituminous binders depends on the type of neat bitumen and the type of modifier, e.g., SBS, SBR, EVA, crumb rubber [15,16]. The process of overheating can be treated as a continuation of the standard technological aging. In the first, natural aging phase, there are logical changes: hardening of the binder, increase of viscosity and softening temperature. However, after reaching a certain temperature (maintained for a certain time, e.g., 1 h), the penetration increases, viscosity decreases, which is illogical. Such an illogical change means that the standard aging process has ended and the destruction of the binder structure has been started (decomposition of the colloidal structure, loss of mass, beginning of the formation of a carbon layer, degradation of the polymer network).

Studies involving asphalt mixtures in an asphalt ignition oven conducted at Purdue University [17,18] showed the complexity of the overheating phenomenon. It was concluded that after the oven temperature exceeds 345 °C, the mixture temperature increases not as a result of the overheating process, but due to burning of the asphalt binder (an exothermic reaction).

Research at the Wuhan University of Technology involved heating non-modified and SBS polymer-modified bitumens in a thermogravimetric (TGA) device and a nitrous environment from room temperature to a temperature of 760 °C, with a constant increment of 10 °C/min [19]. It was concluded that the tested binders exhibited two similar mass loss temperature ranges. The first range of 300–550 °C involved mass loss caused mainly by the oxidation (volatilization) of light oil components (saturated and aromatic), as well as decomposition of the asphaltene system, and in the case of polymer-modified bitumens also the SBS network destruction. The second stage of mass loss involved a range of 550–760 °C and the major cause of this phenomenon was determined to be the process of further oxidation of fading asphaltene residues and the carbonization of the remaining binder components. The research paper [20] describes heating SBS polymer-modified bitumen from room temperature to 700 °C and concludes that the highest mass loss occurred for the range of approximately 360–500 °C. Such high temperatures, however, can occur in the case of emergency situations or random event (e.g., fires in tunnels). However, it does not mean that in the case of the 200–300 °C temperature range there are no processes which could significantly change binder properties. The mass loss phenomenon was identified already for a temperature range of 260–368 °C. The cause for mass loss in this regard was the decomposition of light oil components of bitumen. With the simultaneous impact of thermal radiation and under the presence of oxygen, light-weight components are combusted, volatile substances are formed and a carbon layer begins to form [20].

The scientists from the National University of La Plata-Argentina concluded in their research paper [21] that the binders especially vulnerable to degradation caused by high temperatures were bitumens modified with the SBS polymer. The degradation of pure SBS polymer (without bitumen) was analyzed with the use of the infrared (IR) spectroscopy and chromatography methods. It was concluded that after 30 min of heating the linear polymer at a temperature of 180 °C the copolymer is disintegrated, which results in the formation of products with a larger molecular mass than the molecular mass of the copolymer. Another thermal degradation mechanism occurs in the case of branched block polymer. The consequence of the thermal oxidation processes in the polybutadiene and polystyrene phases are intensive polymer chain splitting or displacement reactions, which results in the formation of new reaction products—free radicals. The same degradation mechanisms occur in the case of polymer-modified bitumen. According to Cortizo et al. [21] the forming polymer free radicals can react and combine with bitumen components with a double C=C bond, which results in increasing polarity and the size of modified bitumen particles (carbonyl and carboxyl). SBS polymer degradation involves the reduction of long and heavy polymer chain into shorter forms, which leads to changes of the pavement mechanical properties and durability. The impact of the bitumen-polymer system at the micro- and nano-structural levels is equally important [22].

The research conducted at Polytechnic Mukah Sarawak-Malaysia [23] on the overheating of bitumen 60/70 involved analyzing the impact of high temperature 175–225 °C for 3 h on the properties of bitumen and asphalt concrete with this bitumen. It was concluded that after exceeding a bitumen heating temperature of 189 °C the properties of the asphalt mixture tested with the Marshall method experienced detrimental changes. For example, bitumen heated in temperatures above 205 °C liquefies and the stability of asphalt concrete used with this bitumen decreases. Binder overheating susceptibility is associated with its sensitivity to ageing.

A research paper by the scientists from the Colas Campus for Sciences and Techniques-France [24] proves that the effect of bitumen rolling thin film oven (RTFO) method ageing at a temperature of 230 °C is bigger than the total effect of bitumen ageing according to the standard RTFO procedure (163 °C) and the pressure aging vessel (PAV) procedure. The outcome of the ageing process are changes in the group composition of bitumen, involving the transformation of a certain part of aromatic oil fractions into resins, and then resins into asphaltenes. A significant increase of the temperature changed the bitumen ageing mechanisms, accelerating the formation of asphaltenes.

The paper (Lolly 2017) [25] shows that increasing the temperature by 15 °C during mixture ageing using the Short-Term Ageing (STA) method does not have a significant impact on mixture properties, whereas doubling the standard STA time (from 4 h to 8 h) greatly influences the bitumen and asphalt mixture.

Based on the studies conducted at the Indian Institute of Technology Roorkee [26] it was concluded that SBS polymer-modified bitumens already exhibit great property deterioration after several hours of storage at a temperature of 210 °C. Polymer degrades, which results in decreasing of the elastic recovery (ER), softening point and parameter G*/sin *δ* associated with the resistance to permanent deformations of asphalt mixtures with this binder.

## 2. Scope and Objectives

The objective of the work presented in this paper was to analyze the phenomenon of road bitumen degradation based on a developed new method for evaluating high-temperature degradation of binders and asphalt mixtures which will enable the determination of the overheating limit temperature for binders and mixtures with this kind of binder. The developed method enabled the evaluation of post-overheating thermal sensitivity of binders and asphalt mixtures, in order to predict the change in the durability of asphalt pavements made from these materials. The viscoelastic properties of a binder deteriorate above the limit temperature.

The first phase of the work involved testing non-modified road bitumens and polymer modified bitumens. The penetration, softening point and viscosity of binders not subject to additional heating and after heating for 1 h at a temperature of 200 °C, 250 °C and 300 °C were determined. The Overheating Degradation Index (ODI) of binders was calculated based on the test results which enabled us to evaluate the sensitivity to overheating of the binders and to determine the overheating limit temperature. The second stage of the study involved testing asphalt mixtures for bridge pavements subjected to an identical heating process, as in the case of binders. MA stiffness was tested with the IT-CY method, as well as stiffness and fatigue strength of AC and SMA mixtures with the 4-point bending beam (4PB) method. The ODI of asphalt mixtures was calculated based on the results of tests regarding the MA stiffness modulus using the IT-CY method. Sensitivity to overheating and overheating limit temperatures determined for asphalt mixtures and binders were compared.

## 3. Materials and Methods

### 3.1. Binders

The study involved 3 non-modified binders, i.e., 50/70, 35/50 and 20/30 and 3 modified, i.e., PMB65/105-60, PMB45/80-55 and PMB25/55-60, which are commonly used for constructing road pavements in central Europe. The binders are of industrial production origin. The typical SBS polymer content in the polymer modified bitumen industrially produced in Poland is about 3–5%. The non-modified bitumens were produced as per standard EN 12591 [27], while the modified bitumens according to standard EN 14023 [28].

### 3.2. Asphalt Mixtures

**Figure 1:**
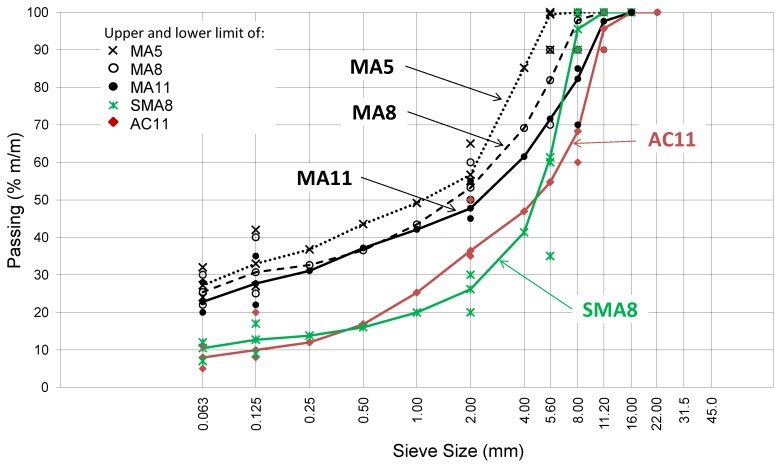
Grading of asphalt mixtures.

Tests regarding five typical mixtures used for wearing and protective courses on bridge facilities were designed and conducted. The mixtures were designed as per the Polish requirements of WT-2 [1]. Mixture grading curves are shown in Figure 1 and the composition and volumetric properties in Table 1.

MA mixtures are used for the protective course, while the SMA and AC mixtures for the wearing course. A typical structure of a bridge pavement consists of a bridge deck insulating course and two courses of asphalt mixtures in a certain arrangement: MA bottom (3–5 cm), AC or SMA top (3–5 cm).

### 3.3. Conventional Testing of Binders

The penetration, softening point and dynamic viscosity of non-modified binders and SBS polymer-modified bitumens were studied. 

The penetration test, which measures the binder consistency in average operating temperatures, was conducted as per standard EN 1426 [29], at a temperature of 25 °C, under a load of 100 g, over a time of 5 s.

The softening point test measures binder consistency in a high operating temperature. It was conducted as per standard EN 1427 [30]. The T_R&B_ temperature was determined, which is a conventional temperature of bitumen transition from the viscoelastic state to a viscous state.

The viscosity test measures binder consistency in high process temperatures. The dynamic viscosity of binders was determined during the study according to standard EN 13302 [31]. The test was conducted in a Brookfield rotational viscometer, and the dynamic viscosity expressed as Pa·s. The test temperatures were adopted: 90 °C, 110 °C and 135 °C, which enabled measuring and comparing the viscosity of binders with various hardness.

### 3.4. Conventional Testing of Asphalt Mixtures

MA stiffness was tested with the IT-CY method, as well as stiffness and fatigue life of AC and SMA with the 4PB method.

The indirect tension specimen stiffness modulus test (indirect tension test on cylindrical specimens IT-CY) was conducted in accordance with standard EN 12697-26 Appendix C [32]. The test was conducted at a temperature of 10 °C.

The research involved studying the impact of overheating of asphalt mixtures on the stiffness and resistance to fatigue cracking (fatigue life) using the 4PB for the average operating temperature range. Prismatic beams with the dimensions of 55 mm wide 55 mm high 400 mm long were cut out from roller compactor compacted plates as per standard EN 12697-33 [33]. The stiffness and fatigue life of mixtures were tested in a hydraulic strength-testing machine equipped with a 4PB bending module. The test involves a 4-point cyclic bending of a beam, with an assumed and constant strain amplitude, changing along a sine function. The stiffness modulus |E*| was determined in accordance with EN 12697-26 Appendix B [32]. A stiffness test involves the determination of load, beam strain, phase angle, number of cycles and calculating the stiffness modulus |E*|. The test is conducted at a temperature of 10 °C, with a strain amplitude of ε = 50 µm/m and frequency of 10 Hz. The stiffness modulus |E*| was determined for a 100th load cycle. 

The fatigue life test was conducted according to standard EN 12697-24 [34]. The test temperature and load frequency are the same as for the 4PB stiffness test. Different strain amplitude values were adopted. For the non-overheated AC asphalt concrete test: 170 µm/m, 200 µm/m, 250 µm/m and 300 µm/m. For AC_250 mixtures (after heating for 1 h at a temperature of 250 °C): 200 µm/m, 225 µm/m and 250 µm/m. For SMA and SMA_250 stone mastic asphalt: 250 µm/m, 300 µm/m, 350 µm/m and 400 µm/m. A specimen fatigue failure was recognized in the case of load, at which the stiffness modulus reached a value equal half (50%) of the initial modulus value, determined during the one-hundredth load cycle. 6 specimens at each strain level were tested. The fatigue characteristic of the mixtures was determined based on fatigue result analysis for different strain levels, using linear regression. 

The coefficient of correlation *r* between fatigue life (number of cycles) and the strain amplitude was calculated in order to evaluate the statistical significance of the correlation. The analysis involved discussing the null hypothesis (lack of difference significance) H_0_: *p* = 0, which means that there is no correlation, and an alternative hypothesis H_1_: *p* ≠ 0, which means that there is a correlation. The parametric *Student-t* test was conducted, the probability of the *t* variable exceeding the limit value was calculated and compared with the significance level *α* = 0.05. If *p* < *α* correlation is statistically significant, then the hypothesis on difference significance is not rejected.

## 4. Overheating

### 4.1. Overheating Method

In order to simulate the impact of high process temperature on binders and asphalt mixtures, a laboratory stand for overheating bituminous materials was developed. The binders and mixtures were subject to high temperatures of 200 °C, 250 °C and 300 °C at a laboratory oven with forced air flow, for one hour. The adopted temperature range (200–300 °C) resulted from an assumption that binder structure failure can occur, which would reveal the parameter value change of an opposite nature in the tested properties of binders or asphalt mixtures with these binders. Analysis of the literature and experience of the authors confirmed that as a result of heating binders and asphalt mixtures to a temperature of about 250 °C, their properties change suddenly. This change occurs either below or slightly above this temperature. For this reason, it was decided to adopt a range where the temperature of 250 °C is in the middle of this range. This made it possible to search for the limit temperature for binder overheating.

The thickness of the heated binder layer was 1.5 mm ± 0.5 mm and about 50 mm ± 5 mm in the case of the asphalt mixture. These materials were overheated in open, steel, large-capacity containers, with provided air access. Appropriate test specimens were formed after the heating process.

### 4.2. Overheating Assessment

The asphalt heating process can be described by the ageing index (IA) presented in a paper by Griffin et al. (1955) [35], with the index being a ratio of bitumen viscosity after being subjected to a temperature of 107 °C for 2 h to a non-aged bitumen. It was also Benson (1976) [35] who suggested an index used to predict asphalt hardening as a result of temperature ageing, in the form of a time, viscosity and penetration function. Liu et al. (2015) [36] determine bitumen ageing degree with the use of the viscosity ageing index (VAI), which utilizes dynamic viscosity at 135 °C before and after ageing. A similar index was applied in a research paper by Radziszewski et al. (2015) [37]. However, these indices do not provide a possibility to easily compare the changes of various binder properties, taking into account temperature increments between different heating levels. They also do not allow us to determine the limit temperature, after exceeding which, the properties change suddenly, not only due to standard ageing mechanisms but also the destructive impact of high temperature. Dessouky (2011) [14] determined the stiffness limit temperature using the Complex Modulus Index (based on PAV or RTFO test). However, these tests applied to standard ageing phenomena. 

In order to better evaluate the effect of overheating binders and asphalt mixtures as well as to compare the changes of different properties caused by the impact of high temperature, a universal Overheating Degradation Index (ODI) was developed, which is calculated as per the following formula:

ODI_Y property_ = (Y_property_ (T_2_) − Y_property_ (T_1_))/Y_property_ (T_1_) (1)

where: ODI_Y property_—Overheating Degradation Index calculated based on Y property readings (e.g., binder penetration and softening point, asphalt mixture stiffness), Y_property_ (T_2_)—value of Y property of the material after heating at temperature T_2_ (e.g., 300 °C), Y_property_ (T_1_)—value of Y property of the material after heating at temperature T_1_, whereas T_1_ < T_2_ (e.g., 250 °C), or value of Y property of the material prior to heating (baseline value).

According to the Formula (1), ODI value is therefore a measure of a change in the analyzed material property between two different heating temperatures or between the heating temperature of the material and the material baseline property-prior to the heating process. To calculate the ODI index, two values of the same Y property should be determined. One value concerns, for example, the penetration of the binder after heating at T_1_ (e.g., 250 °C), the second value refers to penetration of the binder after heating at T_2_ (e.g., 300 °C).

ODIs calculated at several binder or asphalt mixture heating temperature levels can be described by a linear function (trend line), with its inclination (directivity factor) being a measure of material temperature sensitivity to overheating A_(ODI)_. Therefore, the sensitivity to overheating can be expressed as:

A_(ODI)_ = tgα (2)

where: α—angle of inclination of a straight trend line of ODIs determined for various heating temperatures, and the linear function can be expressed in the form:

ODI_Y property_ = A_(ODI)_·x + B (3)

where: A_(ODI)_—directivity factor of linear function, (slope of the function) and it means the tangent of the slope angle and it is a measure of binder or asphalt mixture temperature sensitivity to overheating, x—heating temperature T (e.g., T_1_, T_2_, … T_n_) (°C), B—intersection point of the approximated linear function of ODI with the Y axis (ODI axis).

The slope of the linear function shows how large the change is in the tested property in the assumed temperature range. This is similar to the penetration index (PI) chart. A large change in the same temperature range means a high sensitivity to overheating.

When angle α tends to 0, the tested property is resistant to overheating-induced changes, and when α tends to ±∞, the property is characterized by high sensitivity to overheating. The result of exposure to high temperature can be a failure of binder structure (change in the SARA: Saturates-Aromatics-Resins-Asphaltenes structure, copolymer decomposition, etc.), which can lead to a sudden change of properties, most usually of opposite nature to a change induced by typical ageing processes. In such a case, the ODI value changes from positive (+) to negative (−) or from negative to positive, and the trend line intersects ODI = 0. The point of intersection with the value of 0 enabled reading the overheating limit temperature T_(ODI)_, i.e., the maximum temperature, the exceeding of which means an adverse change of the property caused by failure (temperature degradation) of binder structure or polymer network in modified bitumen. The readings should be made based on several properties in order to determine a reliable temperature T_(ODI)_. The work involved the determination of binder limit temperatures based on the arithmetic mean for the reading of T_(ODI)_ for penetration, softening point and dynamic viscosity tested at 90 °C, 110 °C and 135 °C. Because the intersection of the ODI = 0 value by the trend line (which allows the reading of the limit temperature T_(ODI)_) takes into account a certain “safety margin”, the average T_(ODI)_ better shows the real limit temperature for binder overheating than the reading based on the least favorable result.

A single-factor analysis of variance was used in order to statistically evaluate the significance of differences between the average limit temperature T_(ODI)_ calculated for a group of non-modified bitumens and temperature T_(ODI)_ calculated for a group of modified bitumens (PMB). The analysis involved discussing the null hypothesis (equality of the means) H_0_: *µ*_1_ = *µ*_2_ = … = *µ_n_*, which means that there is no difference between mean values of the dependent variable *µ* and an alternative hypothesis H_1_: *µ_i_* ≠ *µ_j_*, which means that there is a significant difference between at least one pair of different indicators *i*, *j*. The probability *F* of exceeding the critical value was calculated at an assumed level of significance of *p* = 0.05. If *p* < 0.05, the difference between the means is statistically significant and the hypothesis on difference significance is not rejected.

## 5. Results

### 5.1. Binder Overheating

**Figure 2:**
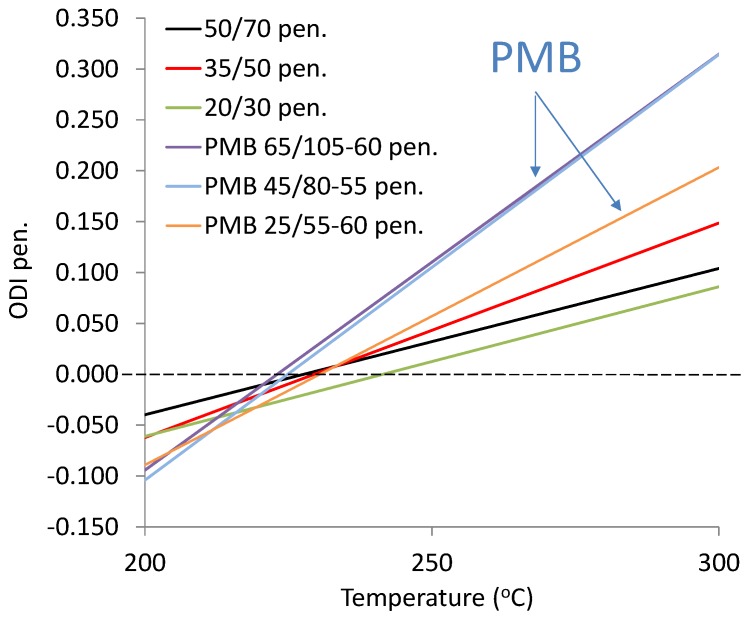
Comparison of sensitivity to overheating A_(ODI)_ of non-modified and modified bitumens, based on an example graph for Overheating Degradation Index of penetration (ODI_pen._).

**Figure 3:**
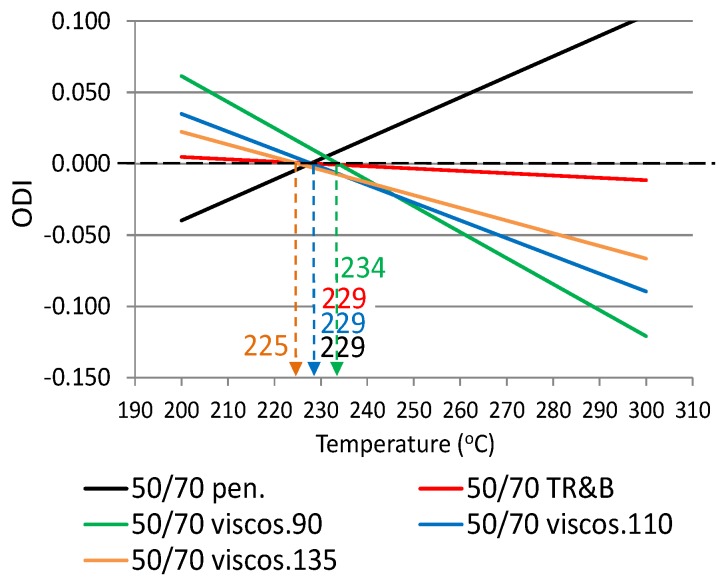
Overheating Degradation Index for binder 50/70 and determination of temperature T_(ODI)_.

**Figure 4:**
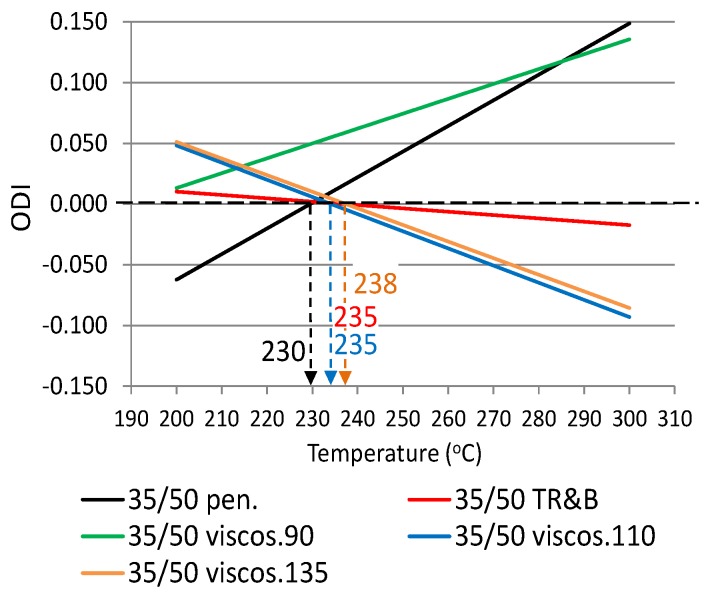
Overheating Degradation Index for binder 35/50 and determination of temperature T_(ODI)_.

**Figure 5:**
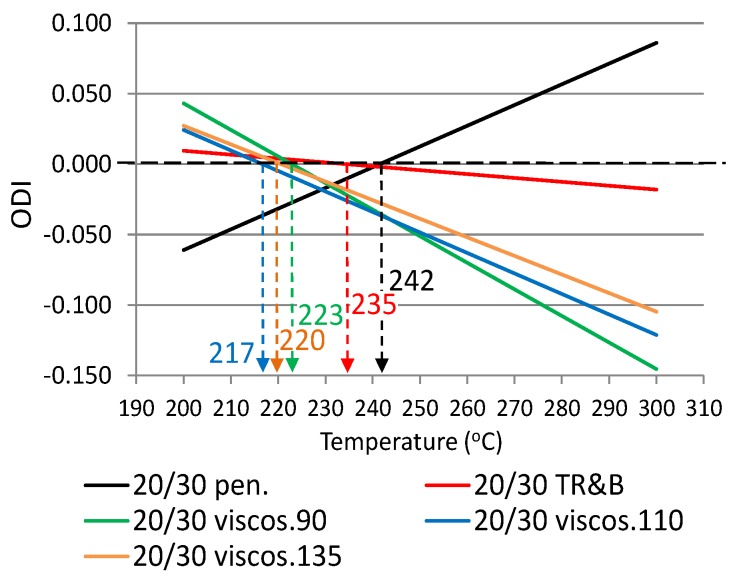
Overheating Degradation Index for binder 20/30 and determination of temperature T_(ODI)_.

**Figure 6:**
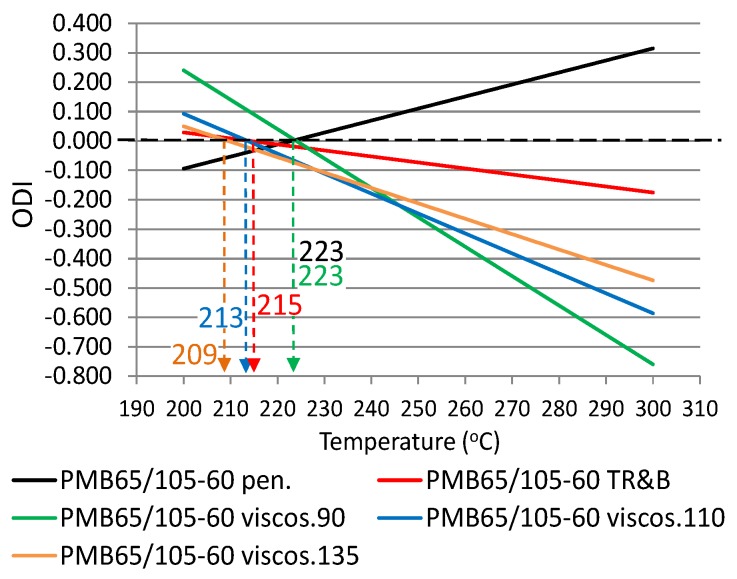
Overheating Degradation Index for PMB65/105-60 and determination of temperature T_(ODI)_.

**Figure 7:**
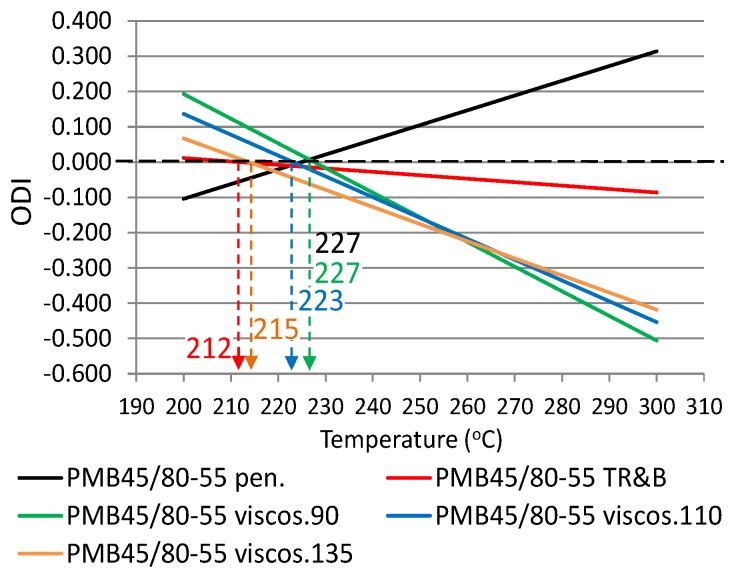
Overheating Degradation Index for binder PMB45/80-55 and determination of temperature T_(ODI)_.

**Figure 8:**
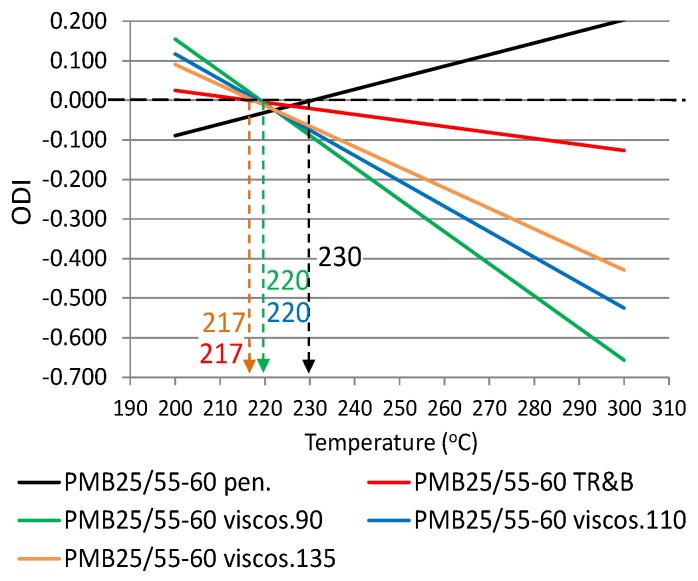
Overheating Degradation Index for binder PMB25/55-60 and determination of temperature T_(ODI)_.

The following ODIs were calculated based on the binder results shown in Table 2: penetration–ODI_pen._, softening point—ODI_TR&B_ and dynamic viscosity—ODI_viscos_. Figure 2 shows an example ODI penetration graph for all analyzed binders in order to compare their sensitivities to overheating A_(ODI)_. A full summary of the ODI graphs, separately for each of the non-modified and modified bitumens, is shown in Figure 3, Figure 4, Figure 5, Figure 6, Figure 7 and Figure 8. The test results regarding bituminous binders before and after heating at three temperatures are shown in Table 2.

When analyzing the ODI pen. results shown in Figure 2 in the form of straight line (trend lines), it should be noted that they are characterized by a varying angle of inclination. The straight line angle of inclination (A_(ODI)_) indicates the rate of change of a tested property, as a result of heating temperature increment within a given range and temperature exposure time. In this case the range was 200–300 °C and the time was 1 h. Therefore, the straight line angle of inclination (A_(ODI)_) is a measure of binder temperature sensitivity to overheating for 1 h. When angle A_(ODI)_ tends to 0, a binder is characterized by the lack of sensitivity to overheating, and when angle A_(ODI)_ tends to ∞, the binder is very sensitive to overheating. Given the above, it can be concluded that the lowest temperature sensitivity is exhibited by road bitumens 50/70 and 20/30, while the highest is exhibited by polymer-modified bitumens PMB45/80-55 and PMB65/105-60. The PMB25/50-60 binder is also characterized by a higher temperature sensitivity compared to non-modified bitumens.

Figure 3, Figure 4, Figure 5, Figure 6, Figure 7 and Figure 8 show ODI results for the tested binders, calculated on the base of penetration and softening point tests with the ring and ball test, as well as dynamic viscosity tested at three temperatures. Then, the limit temperature for binder overheating T_(ODI)_ was calculated at the intersection of a given straight line with ODI = 0. The ultimate limit temperature for a given binder was adopted as an arithmetic mean for individual readings of temperature T_(ODI)_.

**Figure 9:**
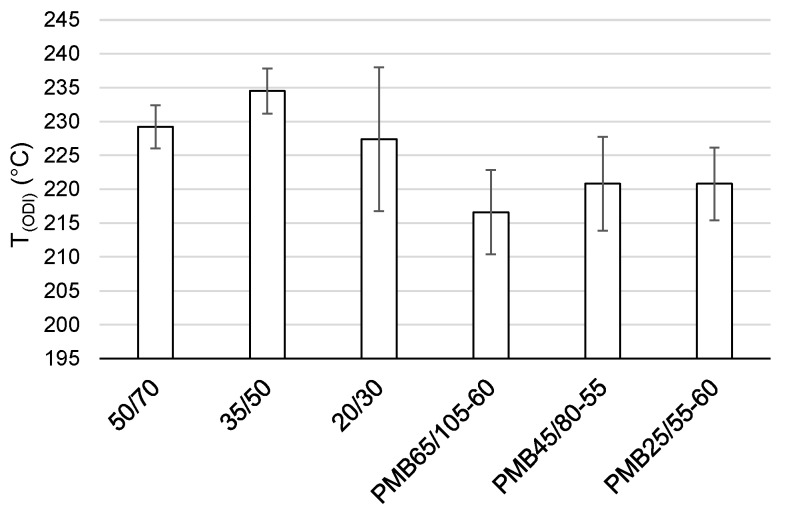
Average T_(ODI)_ for individual binders.

**Figure 10:**
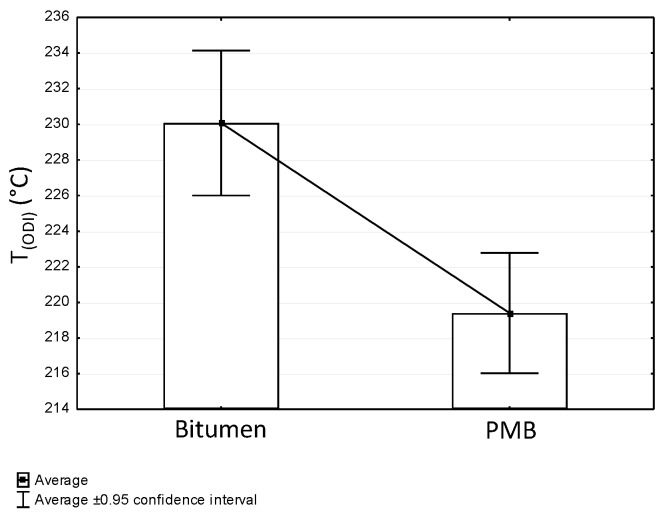
Difference in T_(ODI)_ between road bitumens and polymer modified bitumens (PMB)—ANOVA.

The final summary of the results T_(ODI)_ for a given binder is shown in Table 3 and Figure 9 and Figure 10. Results for which the trend line did not intersect ODI = 0 in a temperature range of 200–300 °C were not taken into account. Such results did not indicate a parameter value change of an opposite nature, which would point to a material structural failure as a result of exposure to high temperature.

When analyzing the test results shown in Figure 3, Figure 4, Figure 5, Figure 6, Figure 7 and Figure 8 and Table 3, it should be concluded that the highest temperature T_(ODI)_ (235 °C) is exhibited by bitumen 35/50, followed by bitumen 50/70 (229 °C). The lowest overheating limit temperature is exhibited by SBS polymer-modified bituminous binders. Temperature T_(ODI)_ of polymer-modifier bitumen PMB 65/105-60 is 217 °C and 221 °C for the other two. It should be concluded that binder property change below the limit temperature is mainly associated with ageing processes (oxidation). After exceeding temperature T_(ODI)_, there is a sudden change of the properties of an opposite tendency (e.g., penetration increase, softening point and viscosity decrease), which indicates bitumen structural failure, and in the case of polymer-modified bitumens, an additional failure of an SBS polymer network.

In order to evaluate the variabilities determined as an arithmetic mean for value T_(ODI)_, the authors calculated standard deviation S_d_, which is shown in Figure 9 in the form of error bars. The coefficient of variant of value T_(ODI)_ was calculated based on standard deviation, as a percentage expressed quotient of S_d_ and the arithmetic mean of T_(ODI)_. As indicated in Table 3 and Figure 9, the highest standard deviation characterizes an bitumen 20/30, followed by a group of polymer-modified bitumens. The coefficient of variation of bitumen 20/30 is 4.69% and is much lower for the other two non-modified bitumens at approximately 1.4%. In the group of modified bitumens, the highest coefficient of variation is exhibited by bitumen PMB45/80-55 (3.14%). Despite the identified differences, it can be concluded that the individual limit temperature values T_(ODI)_ for a given bitumen, taken from the graph, are characterized by little variation.

Figure 10 shows a comparison of the average overheating limit temperature T_(ODI)_ for two basic binder groups, i.e., non-modified and modified bitumens. The ANOVA analysis of variation, with a confidence interval of 95% was applied. Based on the conducted analysis, it was concluded that it was possible to reject the hypothesis regarding the equality of the means at a significance level of p = 0.00017 (*p* < 0.05) and with a probability function F = 19.1, while the difference between binder group was statistically significant.

### 5.2. Asphalt Mixture Overheating

**Figure 11:**
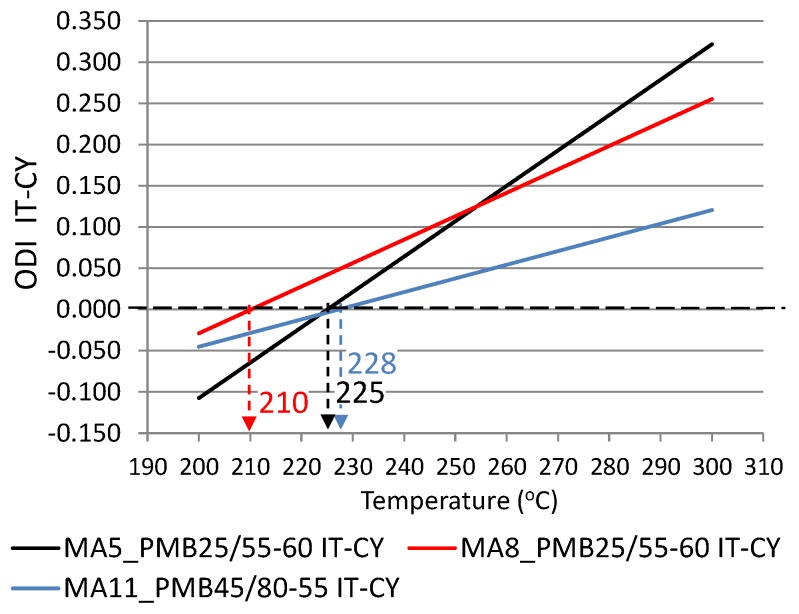
Overheating Degradation Index for IT-CY stiffness of mastic asphalts MA5, MA8 and MA11 with modified bitumens and determination of temperature T_(ODI)_.

**Figure 12:**
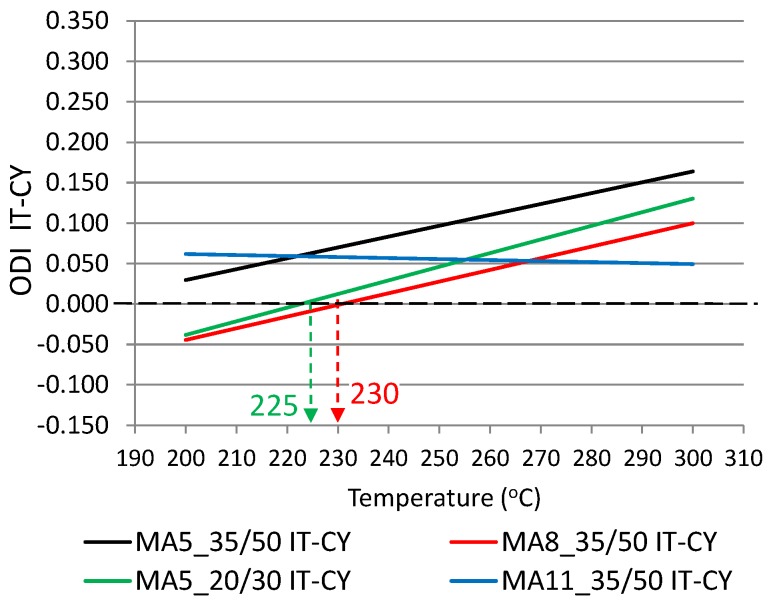
Overheating Degradation Index for IT-CY stiffness of mastic asphalts MA5, MA8 and MA11 with bitumens 35/50 and 20/30 and determination of temperature T_(ODI)_.

The Overheating Degradation Index was calculated based on the results of IT-CY stiffness tests of asphalt mixtures in a heating temperature function, similar to the calculations for binders, as per the Formula (1). Figure 11 and Figure 12 show the results of stiffness modulus IT-CY tests involving mastic asphalts before and after exposure to high temperature and the mixture overheating limit temperatures T_(ODI)_ calculated on the basis of stiffness moduli which are presented in Table 4.

Based on the analysis of the calculated Overheating Degradation Indices for mastic asphalts, it was concluded that the results of stiffness modulus IT-CY test can be used to determine the overheating limit temperature T_(ODI)_ and the temperature sensitivity to overheating A_(ODI)_. As is indicated by Figure 11, the lowest straight line angle of inclination and hence the smallest sensitivity to overheating A_(ODI)_ is exhibited by mastic asphalt MA11 with binder PMB45/80-55. The MA8 mixture is more sensitive, while the MA5 is the most sensitive mixture to overheating, both with the PMB25/55-60 binder. The graph also shows the overheating limit temperature T_(ODI)_, which in the case of mastic asphalt MA11 is 228 °C, and 225 °C and 210 °C for MA5 and MA8 mixtures, respectively. A similar ODI IT-CY for mastic asphalt MA5 and MA8 with non-modified bitumens 35/50 and 20/30 is shown in Figure 12. Both asphalt mixtures with bitumen 35/50 are characterized by a similar sensitivity to overheating A_(ODI)_ (the same straight line angle of inclination). The MA5 mixture with 20/30 bitumen is slightly more sensitive to overheating. The overheating limit temperature for the MA8 mixture with bitumen 35/50 is 230 °C. In the case of mastic asphalt MA5 with the same bitumen, the limit temperature was not read since the straight line did not intersect with ODI = 0. In this case, the stiffness modulus at all heating stages increases and there is no sudden change of the properties exhibiting a reverse nature, which could indicate a structural failure of the composite due to overheating.

**Figure 13:**
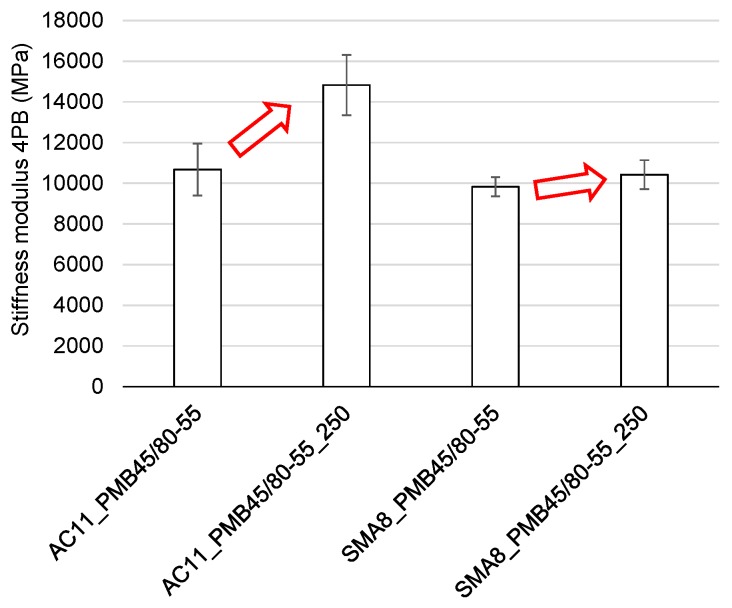
The 4PB stiffness modulus of asphalt concrete AC11 and stone mastic asphalt SMA8 with PMB45/80-55 binder before and after heating for 1h, at a temperature of 250 °C.

**Figure 14:**
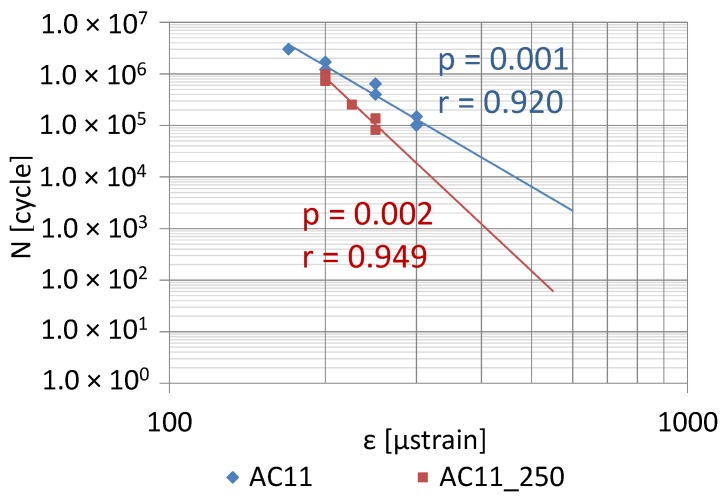
Fatigue life of AC11 mixture with PMB45/80-55, before and after heating at a temperature of 250 °C (1 h).

**Figure 15:**
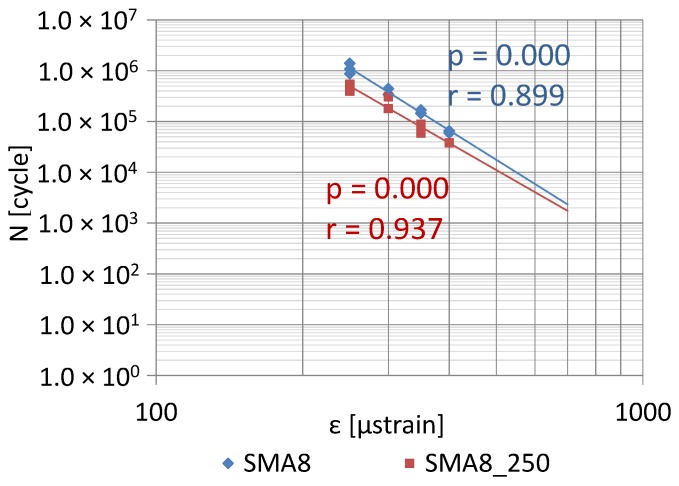
Fatigue life of SMA8 mixture with PMB45/80-55, before and after heating at a temperature of 250 °C (1 h).

The other properties of asphalt mixtures tested to a limit extent due to the time-consuming tests (4PB stiffness and fatigue life) did not enable calculating the ODI and determining the limit temperature T_(ODI)_. However, there are property changes at a temperature of 250 °C, which is shown in Figure 13, Figure 14 and Figure 15.

The stiffness modulus and fatigue life of asphalt mixtures taking into account the impact of a selected high process temperature of 250 °C were conducted with a 4-point bending beam. The test temperature was 10 °C. Figure 13 shows the stiffness modulus test results regarding asphalt concrete and stone mastic asphalt with the same type of polymer-modified bitumen, in the form of arithmetic means for five measurements. Standard deviation S_d_ which was shown in the form of error bars was calculated in order to evaluate the variability of stiffness moduli. The stiffness moduli variation coefficient was calculated based on the standard deviation.

Based on the analysis of the 4PB stiffness modulus tests, it can be noted that at a temperature of 10 °C, the AC is characterized by higher stiffness relative to SMA. After heating at a temperature of 250 °C, the stiffness of the SMA mixture changes slightly (by less than 10%), whereas the stiffness modulus of the AC mixture increased by almost 40%. Moreover, the coefficient of variation of asphalt concrete prior to heating was 11.9%, and 10.0% after heating, while this coefficient for the SMA mixture amounted to 4.8% and 6.7%, respectively. Approximately a twofold smaller dispersion of the stiffness modulus results for the SMA mixture evidences its favorable nature and higher uniformity, both before and after heating at a temperature of 250 °C for 1 h. The cause for this phenomenon is the complexity of a mineral-asphalt composite, the properties of which are impacted by the properties of the binder, grading, the mineral mixture composition and the volumetric properties of the mixture. In the case of mixtures with a more closed structure and with a more developed grit matrix, the impact of the overheating phenomenon on the properties of a mixture is largely reduced.

Figure 14 and Figure 15 show the impact of high temperature on the fatigue life of AC11 and SMA8 with PMB45/80-55 binder. The changes of fatigue life were analyzed using the 4PB method among mixtures before and after heating at a temperature of 250 °C for 1 h.

The correlation statistical analysis was used to conclude that correlations describing fatigue properties of both asphalt mixtures were true (*p* < 0.05). The fatigue life of both mixtures decreased after the heating process. This phenomenon occurs at all of the analyzed strain levels. The fatigue life of the stone mastic asphalt SMA was decreased to a much lesser extent than the fatigue life of asphalt concrete AC. Moreover, the fatigue life of asphalt concrete after heating is reduced faster than the change in the durability of this mixture not exposed to high temperature (higher angle of inclination of the straight line). Such a phenomenon was not observed in the SMA mixture. Therefore, it can be concluded that asphalt concrete is a mixture with higher sensitivity to overheating than SMA with the same binder. Using a binder with a given sensitivity to overheating and a limit temperature T_(ODI)_ is, therefore, not the only factor impacting a change in the properties of asphalt mixtures.

It can be concluded that all mixtures exhibit and adverse nature of property changes caused by bituminous binder overheating, but a correctly selected mixture composition leads to mitigation of this phenomenon.

## 6. Discussion and Conclusions

The test results presented in the article confirm that the process of overheating binders and asphalt mixtures causes adverse changes in their properties, which can significantly decrease pavement durability. Based on the literature study there are different opinions regarding the limit temperature, which can be easily achieved by non-modified and polymer-modified road bitumens through heating, without deteriorating pavement durability. It was shown that the Overheating Degradation Index (ODI) developed within this research paper, together with a presented method for its interpretation, enables the determination of overheating limit temperature T_(ODI)_ and the temperature sensitivity to overheating A_(ODI)_ of non-modified binders and polymer-modified bitumens, as well as asphalt mixtures with these binders. The use of a new formula for the calculation of the ODI reflects a change in the tendency of the tested property, in the form of changing the index mark and reading the limit temperature for binder overheating, which is not possible using the standard aging index. Temperature T_(ODI)_ should be calculated based on several properties, as an arithmetic mean for individual readings. It was shown that in the case of bituminous binders, the necessary temperature can be determined based on testing penetration at 25 °C, the softening point (TR&B) and dynamic viscosity at various temperatures. It is necessary to continue investigating the phenomenon of overheating using other types of binders, modifications and properties on the basis of which the limit temperatures T_(ODI)_ will be calculated. The bitumen modified with a higher SBS content or modified with the EVA polymer also probably shows changes related to damage to the colloidal structure of the base bitumen and the polymer network damage. It is difficult to say unequivocally which type of degradation is dominant, but these types certainly run simultaneously. The research should be continued by assessing the results also according to another methodology, e.g., PG (complex modulus G*, phase angle). In combination with the currently obtained results, they will allow a precise assessment of the temperature degradation of the binder structure. It was concluded that the results of stiffness modulus IT-CY tests can be used in order to calculate ODIs, and then to determine the temperature T_(ODI)_ of asphalt mixtures. An ODI calculated for asphalt mixtures with polymer-modified bitumens clearly shows the temperature at which the tendency of the changes in a studied parameter is reversed. Such a change points to an internal structure failure of the binder and a degradation of the polymer network, due to the impact of excessive temperature. The analysis of the ODI may refer to asphalt mixtures subjected to heating by various methods, e.g., by using microwaves, according to the research [38,39,40,41].

The following conclusion can be obtained from the analysis of the experimental results:

1. Based on the calculated temperature T_(ODI)_ for different European bituminous binders used for road and bridge pavements, it was concluded that non-modified bitumens are more resistant to overheating than polymer-modified bitumens. Variance analysis showed that the difference between these two binder groups was statistically significant (*p* < 0.05). Bitumen 35/50 is characterized by temperature T_(ODI)_ higher by 18 °C than the polymer-modified bitumen PMB 65/105-60. Lower temperature sensitivity of a binder A_(ODI)_ does not always mean a higher limit temperature T_(ODI)_. However, it does shows the rate of change of the studied property, along with increasing heating temperature.
2. Based on the analysis of the stiffness modulus IT-CY tests regarding the mastic asphalt with polymer-modified bitumen PMB25/55-60, it was concluded that the limit temperature T_(ODI)_ for the MA5 mixture was 225 °C, and for MA8 T_(ODI)_ = 210 °C. Comparing these temperatures with the temperature determined for PMB25/55-60 (T_(ODI)_ = 221 °C) it can be stated that there is no direct correlation of the limit temperature determined for a binder and the temperature determined for a asphalt mixture. However, these are similar temperatures. In the case of mastic asphalt MA11 with polymer-modified binder PMB45/80-55, the mixture limit temperature T_(ODI)_ = 228 °C. This temperature is 7 °C higher than the limit temperature for polymer-modified bitumen PMB45/80-55, calculated on the basis of penetration, softening point and viscosity tests (T_(ODI)_ = 221 °C). The limit temperature for a MA8 mixture with bitumen 35/50 was approximately 230 °C, which is a value close to the temperature T_(ODI)_ = 235 °C read for bitumen 35/50. The limit temperature for mastic asphalt MA5 with bitumen 20/30 corresponds to the temperature determined for this bitumen based on penetration, softening point and viscosity tests (T_(ODI)_ = 227 °C).
3. The sensitivity to overheating A_(ODI)_ of asphalt mixtures of the same type but with different binders can be evaluated by comparing the inclination of ODI lines shown in Figure 11 and Figure 12. Sensitivity A_(ODI)_ of both MA5 and MA8 mastic asphalts is higher in the case of mixtures with polymer-modified bitumens, compared to mixtures with non-modified bitumens. Meanwhile the temperature sensitivity of mastic asphalt MA11 with polymer-modified bitumen PMB45/80-55 is the same as of mastic asphalt MA5 with bitumen 20/30. It should be noted that the sensitivity of this polymer-modified bitumen, determined based on penetration at 25 °C, is the highest among the analyzed binders, and the one for bitumen 20/30 is the lowest (Figure 2). This means that the ultimate resistance to overheating of a asphalt mixture is impacted by its grading, composition and volumetric properties, and bitumen temperature sensitivity is only one of the components.
4. As indicated by the conducted tests, the overheating temperature of a mixture with a given binder can differ by ±10 °C from the limit temperature of such a binder. In the case of mixtures with a more closed structure and with a more developed grit matrix, the impact of the overheating phenomenon on the properties of a mixture is largely reduced, despite the application of polymer-modified bitumen. MA11 and SMA8 mixtures are characterized by such resistance.
5. The research paper involved analyzing the impact of heating on changes to the stiffness modulus and fatigue life according to the 4PB method, utilizing asphalt concrete and SMA with polymer-modified bitumen PMB45/80-55, with the temperature T_(ODI)_ = 221 °C. Overheating these mixtures by 30 °C in relation to T_(ODI)_ of the polymer-modified bitumen, resulted in an increase of their stiffness modulus and a significant decrease of their fatigue life. Asphalt concrete turned out to be more sensitive to overheating, and suffered greater adverse changes, leading to the loss of viscous properties in favor of brittle fracture. In the case of unheated mixtures, with the same strain amplitude, e.g., ε = 300 µm/m, the fatigue life of an SMA mixture was about four times higher than that of asphalt concrete. Even greater differences in the resistance to fatigue cracking were observed in the case of mixtures subjected to heating at a temperature of 250 °C. For a strain amplitude ε = 300 µm/m, the fatigue life of an SMA mixture was about ten times higher than that of asphalt concrete. The significance of identified relationships was confirmed using the statistical methods.

## Figures and Tables

**Table 1:** Composition and volumetric properties of asphalt mixtures.

Properties	Units	MA 5	MA 8	MA 11	SMA 8	AC 11
Lime filler content	%	29.4	27.6	25.0	11.2	8.5
Fine aggregate content	%	20.2	22.1	18.6	12.1	28.4
Coarse aggregate content (gabbro)	%	42.4	42.3	48.9	69.7	57.7
Binder content	%	8.0	8.0	7.5	7.0	5.4
Air voids content V_a_ (blows: 2 × 75–AC; 2 × 50–SMA)	%	0.4	0.1	0.2	2.6	2.1
Voids in mineral aggregate VMA	%	17.6	18.5	18.6	19.8	15.6
Voids filled with bitumen VFB	%	97.8	99.4	99.1	86.9	86.6

**Table 2:** The properties of non-modified and SBS polymer-modified bituminous binders before and after heating at a high temperature (1 h).

Type of Bitumen	Properties	Units	Heating Temperature (°C)			
			-	200	250	300
50/70	Penetration, 25 °C	(0.1 mm)	57.1	56.7	54.8	62.3
	Softening point	(°C)	48.9	48.9	49.2	48.4
	Viscosity, 90 °C	(Pa·s)	9.06	9.72	9.21	8.20
	Viscosity, 110 °C	(Pa·s)	1.92	1.99	1.93	1.76
	Viscosity, 135 °C	(Pa·s)	0.45	0.46	0.45	0.42
35/50	Penetration, 25 °C	(0.1 mm)	37.3	36.7	34.9	41.7
	Softening point	(°C)	54.3	54.7	54.8	53.7
	Viscosity, 90 °C	(Pa·s)	18.60	19.21	19.88	22.97
	Viscosity, 110 °C	(Pa·s)	3.38	3.51	3.50	3.14
	Viscosity, 135 °C	(Pa·s)	0.70	0.72	0.74	0.66
20/30	Penetration, 25 °C	(0.1 mm)	24.5	23.0	23.3	25.3
	Softening point	(°C)	61.4	61.7	62.0	60.6
	Viscosity, 90 °C	(Pa·s)	49.44	51.11	49.45	41.8
	Viscosity, 110 °C	(Pa·s)	7.48	7.46	7.5	6.39
	Viscosity, 135 °C	(Pa·s)	1.29	1.31	1.29	1.14
PMB65/105-60	Penetration, 25 °C	(0.1 mm)	64.7	61.1	63.1	85.4
	Softening point	(°C)	60.6	59.8	60.5	47.3
	Viscosity, 90 °C	(Pa·s)	55.56	64.50	58.00	9.29
	Viscosity, 110 °C	(Pa·s)	6.98	6.96	6.58	2.09
	Viscosity, 135 °C	(Pa·s)	1.25	1.23	1.13	0.52
PMB45/80-55	Penetration, 25 °C	(0.1 mm)	46.2	41.3	45.8	60.1
	Softening point	(°C)	56.6	56.0	56.4	50.3
	Viscosity, 90 °C	(Pa·s)	28.78	30.36	33.96	12.09
	Viscosity, 110 °C	(Pa·s)	5.06	5.33	5.37	2.49
	Viscosity, 135 °C	(Pa·s)	1.14	1.16	1.07	0.57
PMB25/55-60	Penetration, 25 °C	(0.1 mm)	32.2	29.2	31.1	37.3
	Softening point	(°C)	67.0	67.2	66.7	56.8
	Viscosity, 90 °C	(Pa·s)	110.13	115.0	111.63	25.97
	Viscosity, 110 °C	(Pa·s)	12.20	12.40	12.38	4.63
	Viscosity, 135 °C	(Pa·s)	1.86	1.89	1.85	0.92

**Table 3:** Overheating limit temperatures T_(ODI)_ for modified and non-modified bituminous binders, before and after heating at a high temperature (1 h).

Type of Bitumen	Fitting Formula of ODI ODI_Y property_ = A_(ODI)_x + B	T_(ODI)_ (°C)	Average T_(ODI)_ (°C)	Sd (°C)
50/70	ODI_pen_ = 0.0014x − 0.3276	229	229	3.19
	ODI_R&B_ = −0.0002x + 0.0373	229		
	ODI_viscos (90)_ = −0.0018x + 0.4265	234		
	ODI_viscos (110)_ = −0.0012x + 0.2841	229		
	ODI_viscos (135)_ = −0.0009x + 0.2002	225		
35/50	ODI_pen_ = 0.0021x − 0.4841	230	235	3.32
	ODI_R&B_ = −0.0003x + 0.0650	235		
	ODI_viscos (110)_ = −0.0014x + 0.3309	235		
	ODI_viscos (135)_ = −0.0014x + 0.3244	238		
20/30	ODI_pen_ = 0.0015x − 0.3551	242	227	10.64
	ODI_R&B_ = −0.0003x + 0.0644	235		
	ODI_viscos (90)_ = −0.0019x + 0.4201	223		
	ODI_viscos (110)_ = −0.0015x + 0.3149	217		
	ODI_viscos (135)_ = −0.0013x + 0.2908	220		
PMB65/105-60	ODI_pen_ = 0.0041x - 0.9125	223	217	6.23
	ODI_R&B_ = −0.002x + 0.4392	215		
	ODI_viscos (90)_ = −0.01x + 2.2419	223		
	ODI_viscos (110)_ = −0.0068x + 1.4522	213		
	ODI_viscos (135)_ = −0.0052x + 1.0972	209		
PMB45/80-55	ODI_pen_ = 0.0042x − 0.9407	227	221	6.94
	ODI_R&B_ = −0.001x + 0.2067	212		
	ODI_viscos (90)_ = −0.007x + 1.5904	227		
	ODI_viscos (110)_ = −0.0059x + 1.3157	223		
	ODI_viscos (135)_ = −0.0048x + 1.0363	215		
PMB25/55-60	ODI_pen_ = 0.0029x − 0.6742	230	221	5.36
	ODI_R&B_ = −0.0015x + 0.3276	217		
	ODI_viscos (90)_ = −0.0081x + 1.7781	220		
	ODI_viscos (110)_ = −0.0064x + 1.4023	220		
	ODI_viscos (135)_ = −0.0052x + 1.1278	217		

**Table 4:** IT-CY stiffness modulus and overheating limit temperatures T_(ODI)_ for mastic asphalt with modified and non-modified binders, before and after heating at a high temperature (1 h).

Asphalt Mixture	Type of Binder	Stiffness Modulus IT-CY (MPa)				Fitting Formula of ODI_IT-CY_ (ODI_Y property_ = A_(ODI)_x + B)	T_(ODI)_ (°C)
		Heating Temperature (°C)					
		-	200	250	300		
MA5	35/50	5603	5640	6443	7352	ODI_IT-CY_ = 0.0013x − 0.2395	- *
	20/30	8399	7520	8867	9434	ODI_IT-CY_ = 0.0017x − 0.3754	**225**
	PMB25/55-60	5408	4842	5333	7064	ODI_IT-CY_ = 0.0043x − 0.9660	**225**
MA8	35/50	6168	5774	6158	6653	ODI_IT-CY_ = 0.0014x − 0.3330	**230**
	PMB25/55-60	5945	5771	6429	8067	ODI_IT-CY_ = 0.0028x − 0.5970	**210**
MA11	35/50	6478	7076	7040	7601	ODI_IT-CY_ = -0.0001x + 0.0872	- *
	PMB45/80-55	4958	4490	5103	5466	ODI_IT-CY_ = 0.0017x − 0.3761	**228**

* the nature of property changes does not indicate a reversed trend.
